# A late bearing insert dislocation was associated with new-onset rheumatoid arthritis after unicompartmental knee arthroplasty: a rare case report

**DOI:** 10.1186/s12891-026-09840-8

**Published:** 2026-04-20

**Authors:** Huan Liu, Bo Wu

**Affiliations:** Department of Orthopaedics, The 960th Hospital of PLA, 25 shifan Road, Tianqiao District, Jinan, Shandong 250031 China

**Keywords:** Rheumatoid arthritis, Mobile-bearing unicompartmental knee arthroplasty, Bearing dislocation, Case report

## Abstract

**Background:**

Bearing dislocation is a unique complication of mobile-bearing unicompartmental knee arthroplasty (UKA), with diverse underlying mechanisms. This article reports a rare mechanism of bearing dislocation following mobile-bearing UKA, which was probably associated with the new-onset rheumatoid arthritis (RA) leading to anterior cruciate ligament (ACL) insufficiency—aiming to highlight this uncommon etiology and its implications for clinical practice.

**Case presentation:**

A 53-year-old woman presented with acute right knee pain and restricted mobility one day prior to admission, who was treated by mobile-bearing UKA for anteromedial osteoarthritis two years ago. Radiographs revealed bearing dislocation in this patient. Laboratory evaluation revealed elevated inflammatory markers (CRP 71.59 mg/L, ESR 74 mm/h) and positive RA serology(anti-CCP 39.09 U/mL, RF 32.4 IU/mL). Periprosthetic joint infection was rigorously excluded through multiple methods. The patient responded well to short-term corticosteroids and underwent one-stage revision total knee arthroplasty. Intraoperatively, extensive chronic synovitis, bearing dislocation, and severe ACL erosion/laxity due to synovial proliferation were observed. Histopathology confirmed synovial hyperplasia with dense lymphoplasmacytic infiltration and lymphoid follicle formation. Following standardized anti-rheumatic therapy, the patient regained satisfactory knee function with normalized inflammatory markers at 12-month follow-up.

**Conclusion:**

This case highlights that in patients who was present with delayed pain and elevated inflammatory markers after UKA. Inflammatory joint diseases such as RA should be included in the differential diagnosis alongside infection. Appropriate serological screening and multidisciplinary collaboration with rheumatology are essential for optimal management. As a single case report, these findings are hypothesis-generating and warrant confirmation in larger studies.

## Background

Mobile-bearing unicompartmental knee arthroplasty (UKA) is a key surgical option for treating anteromedial knee osteoarthritis, as it preserves knee proprioception and most physiological motion, making it a joint-preserving procedure [1]. Bearing dislocation is a unique complication of this technique, with reported incidence rates varying geographically. In some East Asian countries, the incidence is reported approximately 2.37% [2]. Early dislocation is typically associated with surgical technical factors, including component malposition, inadequate ligament balancing, bearing impingement, or incorrect bearing size selection [3, 4]. Late dislocation is more often attributed to polyethylene wear, progressive ligament attenuation, component loosening [5].

Rheumatoid arthritis (RA) is a systemic autoimmune disease characterized by aggressive synovitis that can progressively erode cartilage, bone, and periarticular soft tissues [6]. Historically, RA has been considered a contraindication for the UKA. This recommendation stems from the fundamental design principle articulated by Goodfellow and O’Connor: the UKA relies heavily on intact ligament function—particularly the anterior cruciate ligament (ACL)—to guide femoral rollback and maintain bearing stability [7]. Since RA typically involves multiple knee compartments and can compromise ligament integrity, it was deemed unsuitable for this procedure [8]. However, recent evidence has begun to challenge this absolute contraindication. Studies have shown that unicompartmental knee arthroplasty can achieve acceptable mid-term outcomes in carefully selected patients with rheumatoid arthritis whose disease is well-controlled (treated to target with disease-modifying antirheumatic drugs) [9].

**Fig. 1:**
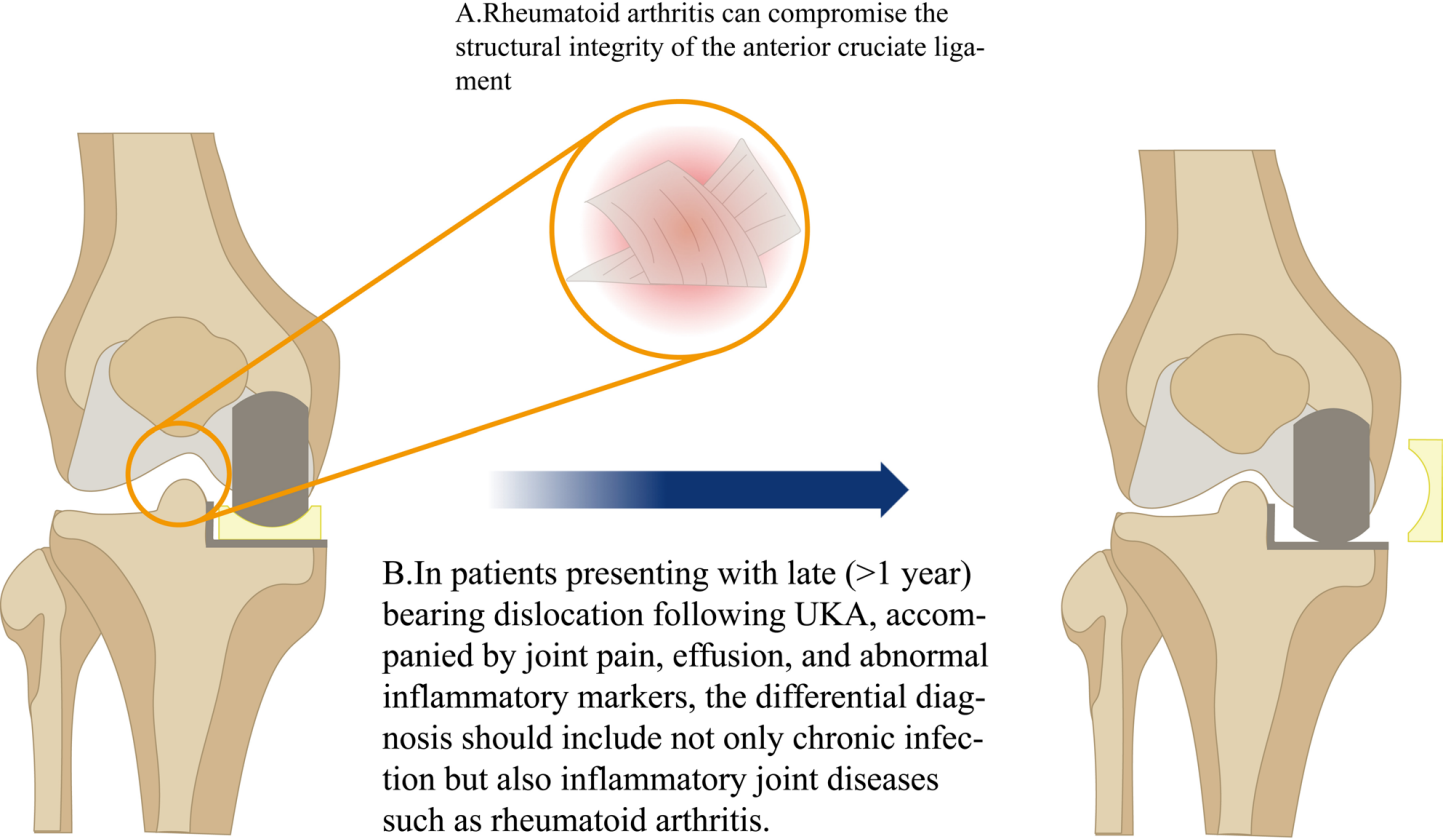
Key Clinical and Decision-making Considerations in the Management of Late Bearing Dislocation Following UKA. **A** Rheumatoid arthritis can compromise the structural integrity of the anterior cruciate ligament. **B **Differential diagnosis for late bearing dislocation should include both infection and inflammatory joint diseases such as rheumatoid arthritis

The case we present introduces a mechanism not previously well documented, which was probably associated with the new-onset RA after a successful UKA, leading to inflammatory erosion of the ACL and subsequent bearing dislocation(Fig. 1). This article reports this rare mechanism in detail, presenting the diagnostic process, intraoperative findings, and multidisciplinary management. To further enhance our understanding of the mechanisms of late dislocation, we aim to highlight the importance of considering inflammatory joint disease in the differential diagnosis of late UKA failure.

## Case presentation

### History of illness

At the time of primary UKA, the patient was a 51-year-old woman with a BMI of 26.4 kg/m². She had no significant comorbidities (hypertension, diabetes, cardiovascular disease, or autoimmune conditions), no regular medications, and a moderate activity level.

**Fig. 2:**
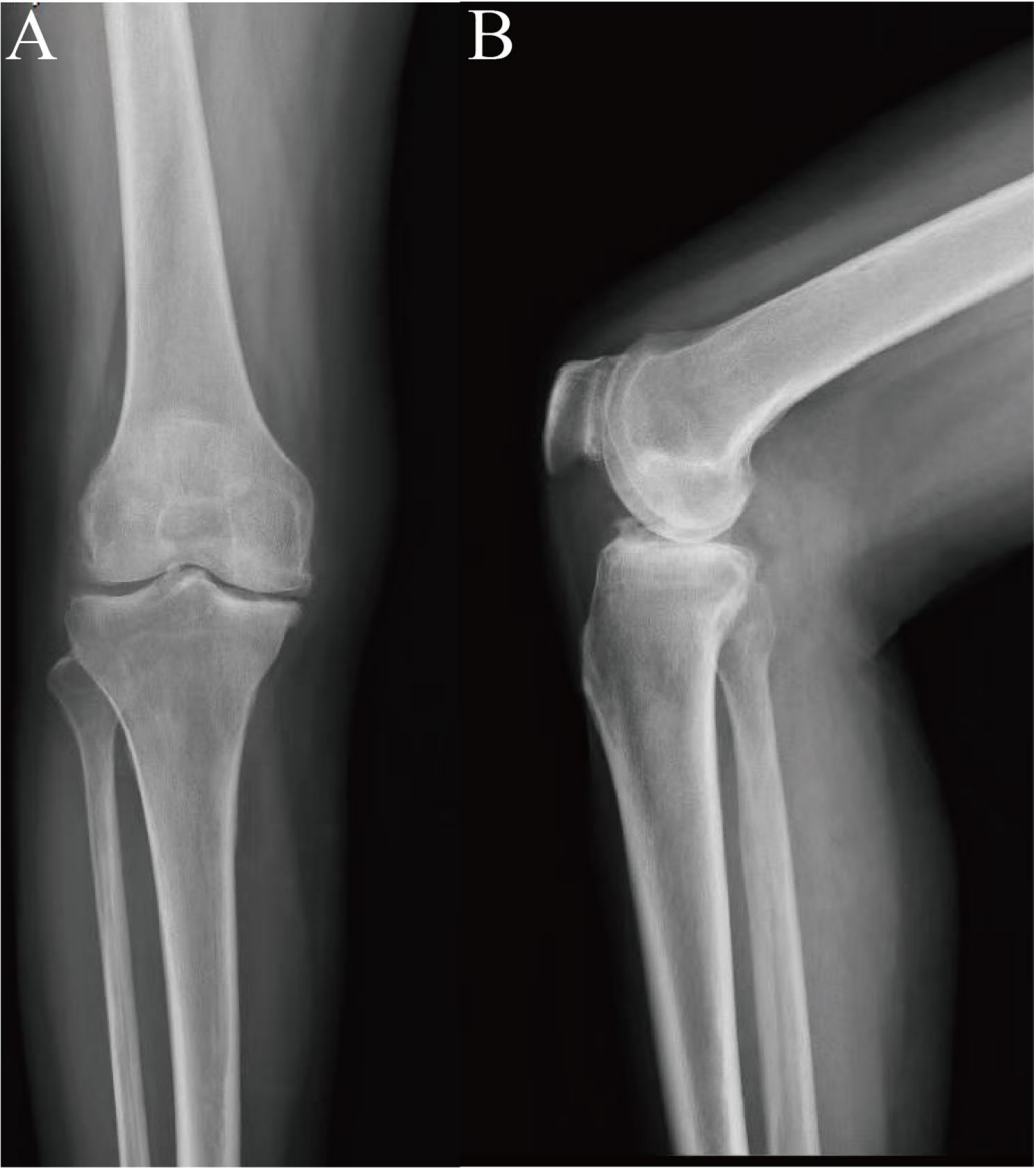
Anteroposterior and lateral radiographs of the knee before unicompartmental knee arthroplasty (**A** and **B**). Narrowing of the knee joint space and sclerosis of the articular surfaces (**A** and **B**)

She had undergone stardard UKA for the anteromedial osteoarthritis of the right knee two years ago (Fig. 2). The index UKA was an Oxford^®^ Phase 3 mobile-bearing prosthesis (Zimmer Biomet) with a size medium femoral component, size C tibial component, and a 4 mm polyethylene bearing. 

During the initial treatment, primary results of inflammatory markers were proven normal (CRP < 3.19 mg/L, ESR 2 mm/h) before the operation. The status of ACL was comfirmed intact and functional by the direct probing and visual inspection during the operation. Following the initial surgery, the patient achieved pain-free knee with satisfactory function. Postoperative radiographs demonstrated well-positioned prosthesis with no evidence of loosening (Fig. 2).

**Fig. 3:**
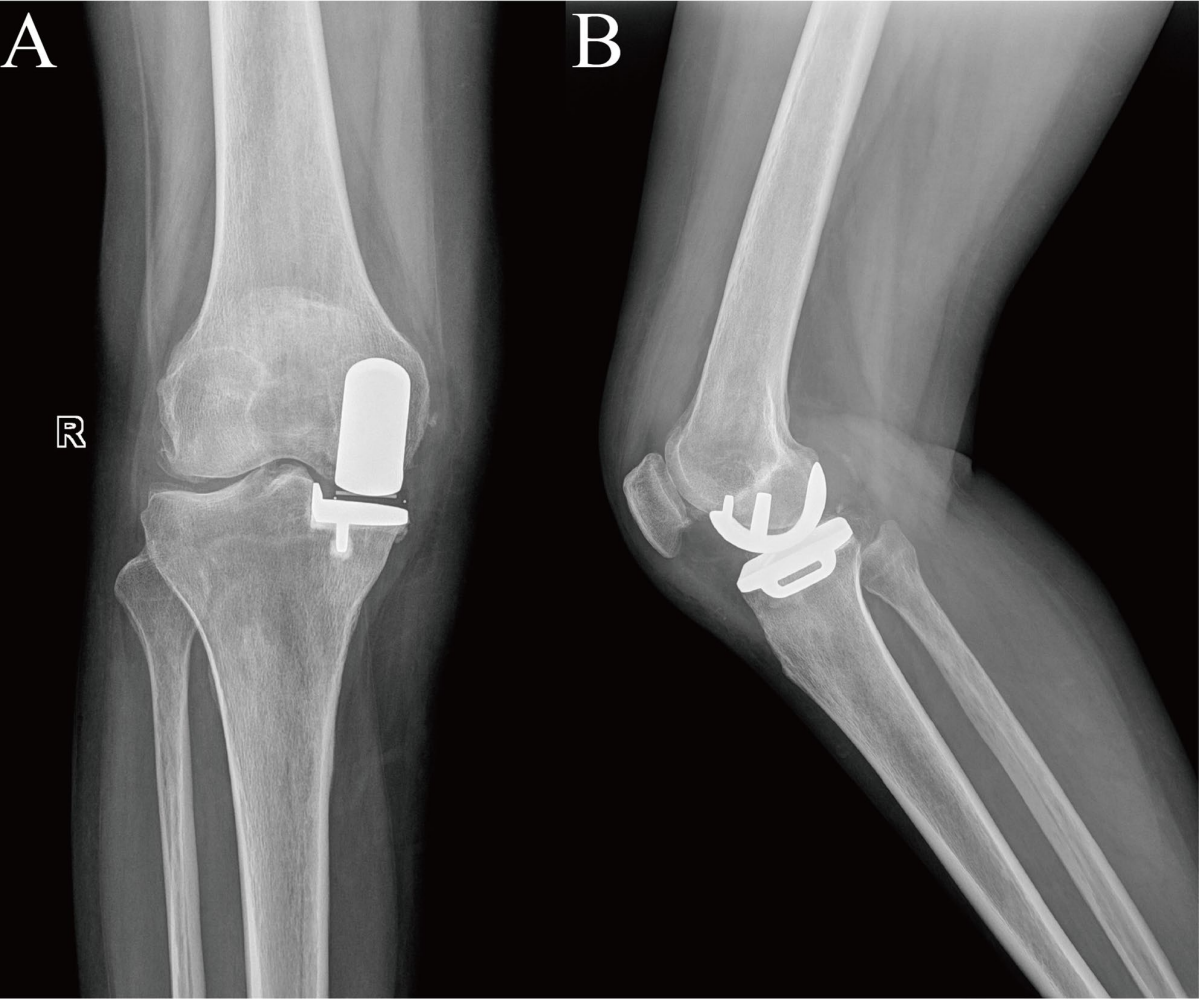
Postoperative anteroposterior and lateral radiographs of the right knee following unicompartmental knee arthroplasty. **A** Anteroposterior view shows the medial unicompartmental prosthesis in the right knee is well positioned with satisfactory alignment. **B** Lateral view demonstrates balanced flexion-extension gaps and secure fixation of the components, with no evidence of loosening or migration

Upon further detailed history-taking, the patient recalled intermittent right knee swelling over the preceding several months and occasional mild pain in both wrists and finger joints without typical morning stiffness. As the absence of significant pain, the patient did not receive any treatment prior to this admission.

On the day prior to admission, the patient experienced sudden-onset acute pain in the right knee with associated restricted mobility. Physical examination revealed a well-healed surgical incision, notable swelling of the suprapatellar pouch, and localized tenderness over the medial joint line. Active and passive range of motion (ROM) was limited to 20°~80°. Ligamentous stress tests and the anterior drawer test could not be reliably assessed due to pain and muscle guarding.

Radiographic evaluation demonstrated displacement of the polyethylene bearing into medial joint space. Additionally, radiolucent lines along the periphery of the tibial component and narrowing of the lateral joint space were evidently observed on radiographs(Fig. 3).

**Fig. 4:**
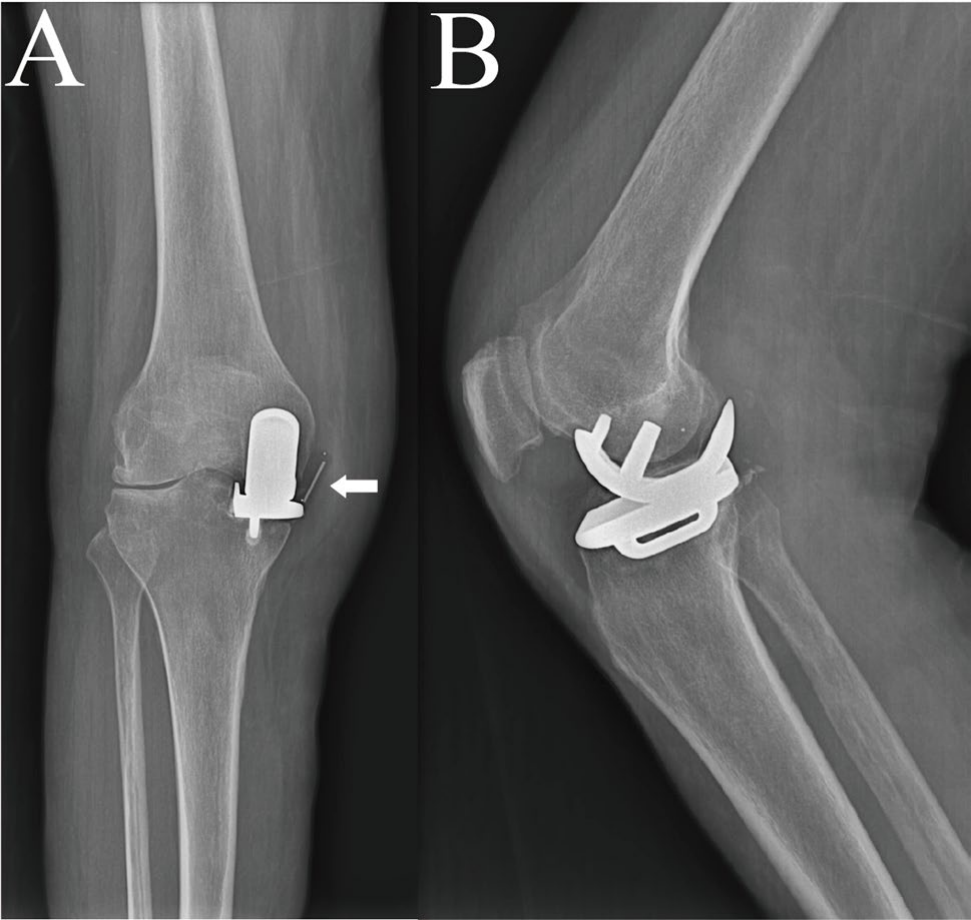
Postoperative radiographs of the right knee following unicompartmental knee arthroplasty. **A **Anteroposterior view: Displacement of the polyethylene bearing into the medial joint space is indicated by the white arrow. Noticeable radiolucent lines are observed along the periphery of the tibial component. **B** Lateral view: Abnormal positioning of the implant is evident

### Infection exclusion

Laboratory tests on admission revealed elevated inflammatory markers: C-reactive protein (CRP) 71.59 mg/L and erythrocyte sedimentation rate (ESR) 74 mm/h.

To rigorously exclude periprosthetic joint infection(PJI), a comprehensive diagnostic workup was performed. Serum procalcitonin was normal (0.03 ng/mL), and both Brucella agglutination test and T-SPOT.TB were negative, reducing the likelihood of atypical or mycobacterial infection.

Diagnostic arthrocentesis yielded slightly turbid, pale-yellow synovial fluid containing multiple white flocculent particles. Gram stain showed moderate white blood cells (6–20 per high-power field) with no organisms seen, and no fungal spores or hyphae were observed.

Synovial fluid was cultured routinely for 7 days and, given the clinical suspicion, incubation was extended to 14 days under both aerobic and anaerobic conditions. All cultures showed no growth of pathogenic bacteria. The patient had not received any antibiotics in the 4 weeks prior to arthrocentesis, confirmed by detailed medication history, eliminating the possibility of culture-negative PJI due to antibiotic suppression.

The combination of prolonged negative cultures, normal procalcitonin, absence of preoperative antibiotics, and negative Gram stain collectively provides strong evidence against infection.

### Rheumatologic evaluation

Based on the history of polyarticular symptoms, the decision to pursue rheumatologic testing was prompted. Rheumatologic antibody testing revealed positive results for anti-cyclic citrullinated peptide (anti-CCP) antibody at 39.09 U/mL and rheumatoid factor (RF) at 32.4 IU/mL. Collectively, these findings shew the patient scored 8 points, confirming the diagnosis of RA, based on the 2010 ACR/EULAR classification criteria for rheumatoid arthritis (threshold for definite RA: ≥6 points).

Given high suspicion of active RA, anti-inflammatory therapy with corticosteroids was initiated. The patient demonstrated a favorable therapeutic response, with reported alleviation of symptoms in the involved joints. Repeat laboratory testing showed improvement in inflammatory markers: CRP decreased from 71.59 mg/L to 10.91 mg/L, and ESR decreased from 74 mm/h to 63 mm/h. This positive response to corticosteroid therapy further supported the diagnosis of RA.

Therefore, the preoperative diagnosis was established as:1.Bearing dislocation following UKA; 2.Rheumatoid arthritis.

After thorough multidisciplinary discussion involving orthopaedics and rheumatology, and with informed consent from the patient, revision surgery was planned.

### Surgical details

#### Intraoperative findings

Upon arthrotomy, the joint cavity contained a moderate amount of clear, pale-yellow synovial fluid with visible white flocculent particles. The articular surfaces were extensively covered by hypertrophic synovial pannus, and the synovium was diffusely hyperplastic, congested, and thickened, with areas of fibrinous exudate deposition—these findings consistent with active inflammatory synovitis. The polyethylene bearing was found displaced beneath the MCL, confirming the preoperative diagnosis of dislocation.

#### Assessment of stabilizing structures

The intercondylar notch was filled with proliferative inflammatory synovium that completely encased and infiltrated the ACL. The ligament was substantially attenuated with marked laxity and complete loss of tension, as confirmed by direct probe palpation and a positive intraoperative anterior drawer test (Fig. 4). These findings provided direct visual evidence of the proposed pathomechanism: inflammatory erosion of the ACL by rheumatoid synovitis leading to ligamentous insufficiency and subsequent bearing dislocation. The collateral ligament was assessed and found to be intact with normal tension on stress testing. Both the femoral and tibial components were assessed for stability and found to be well-fixed without evidence of loosening.

**Fig. 5:**
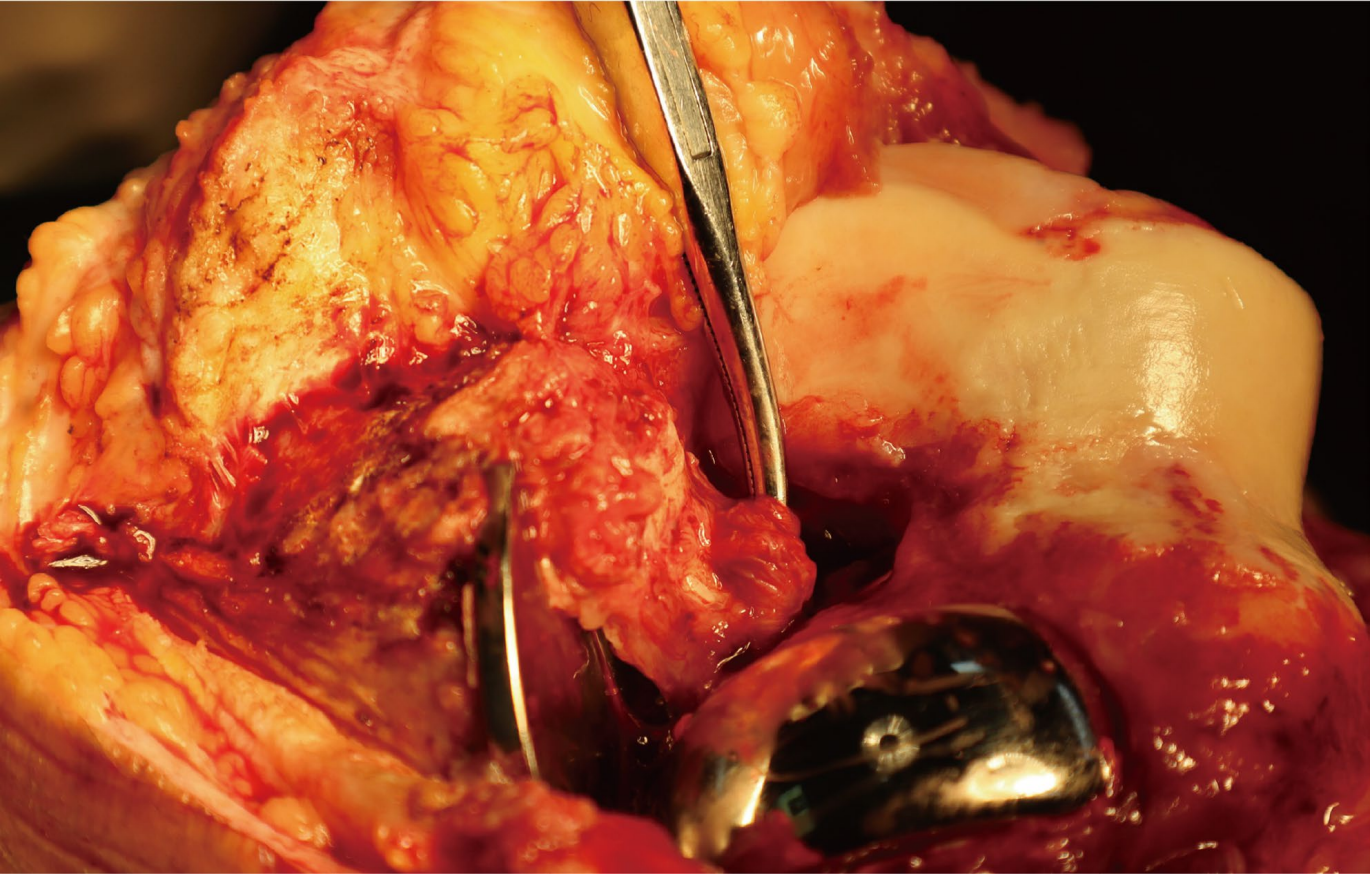
Intraoperative macroscopic view of the right knee.The intercondylar notch is filled with proliferative inflammatory synovium, which completely encases and infiltrates the anterior cruciate ligament (ACL). The ACL is markedly attenuated, lax, and devoid of tension, as confirmed by direct probe palpation and a positive intraoperative anterior drawer test

#### Revision procedure

The revision was performed as a single-stage exchange. The tibial and femoral components were carefully removed with minimal loss of host bone. A thorough synovectomy was performed to debride as much inflammatory tissue as possible. Given the intraoperative confirmation of ACL insufficiency and the underlying diagnosis of active rheumatoid arthritis, a posterior-stabilized total knee arthroplasty (PS-TKA) design (NexGen^®^ LPS, Zimmer Biomet) was selected for revision. Based on intraoperative measurements and trial fitting, the decision was finally made to use a size 7 femoral component, a size 4 tibial baseplate, and to implant a 14 mm-thick polyethylene insert. The components were implanted with standard cemented fixation. The patella was not resurfaced as the articular cartilage was well-preserved.

### Postoperative pathology and culture

Histopathological examination of the synovial tissue revealed synovial hyperplasia with dense interstitial infiltration of lymphocytes and plasma cells, along with lymphoid follicle formation (Fig. 5)—the findings confirming inflammatory arthritis and consistent with rheumatoid arthritis.

**Fig. 6:**
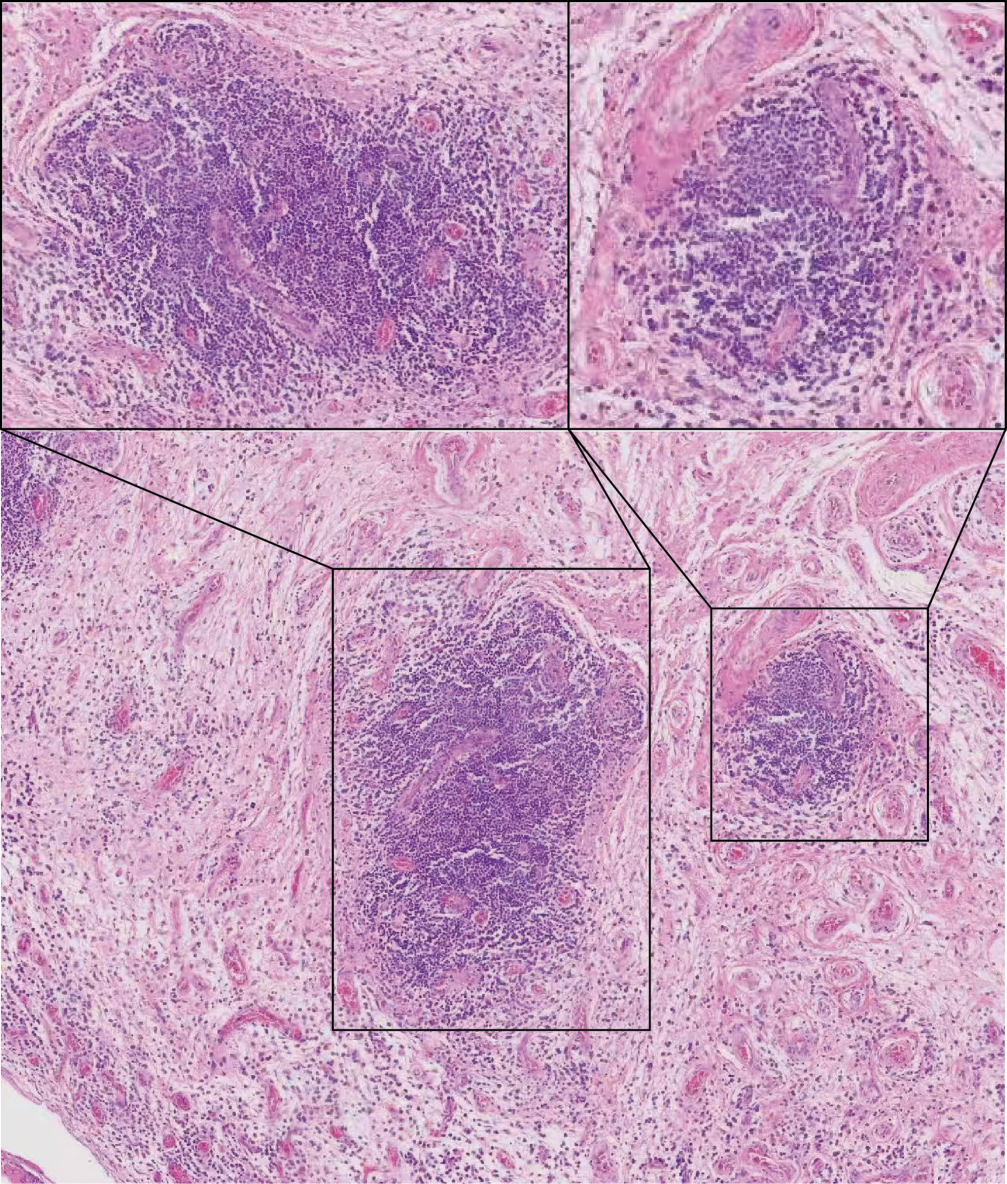
Histopathology of the synovial tissue.Synovial hyperplasia with dense lymphoplasmacytic infiltration and lymphoid follicle formation is observed (high-magnification insets), consistent with the features of rheumatoid arthritis

The intraoperative cultures (tissue and synovial fluid) were obtained prior to antibiotic administration. The results of all cultures subsequently also showed no growth of pathogenic bacteria.

### Postoperative management and follow-up

Postoperatively, the patient was managed jointly by the orthopaedic and rheumatology teams. The rheumatologic treatment was anti-rheumatic therapy of modern disease-modifying anti-rheumatic drugs (DMARDs), including methotrexate, sulfasalazine, and leflunomide.

The patient followed a structured, phased rehabilitation program under the guidance of a physical therapist. At 12-month follow-up, the patient demonstrated favorable clinical and functional outcomes. She reported no pain in the right knee at rest or during activities of daily living, and the joint was clinically stable with no episodes of giving way. Active and passive range of motion was 0°–120°, allowing for normal gait and return to activities of daily living.

Inflammatory markers had normalized, confirming well-controlled rheumatoid arthritis. RF improved to 11 IU/mL, CRP to < 3.06 mg/L, and ESR to 11 mm/h. Follow-up radiographs showed well-positioned revision TKA components with satisfactory alignment and no abnormalities at the prosthesis-bone interface(Fig. 6). The Oxford Knee Score (OKS) was 16/60 points, indicating good knee function.

**Fig. 7:**
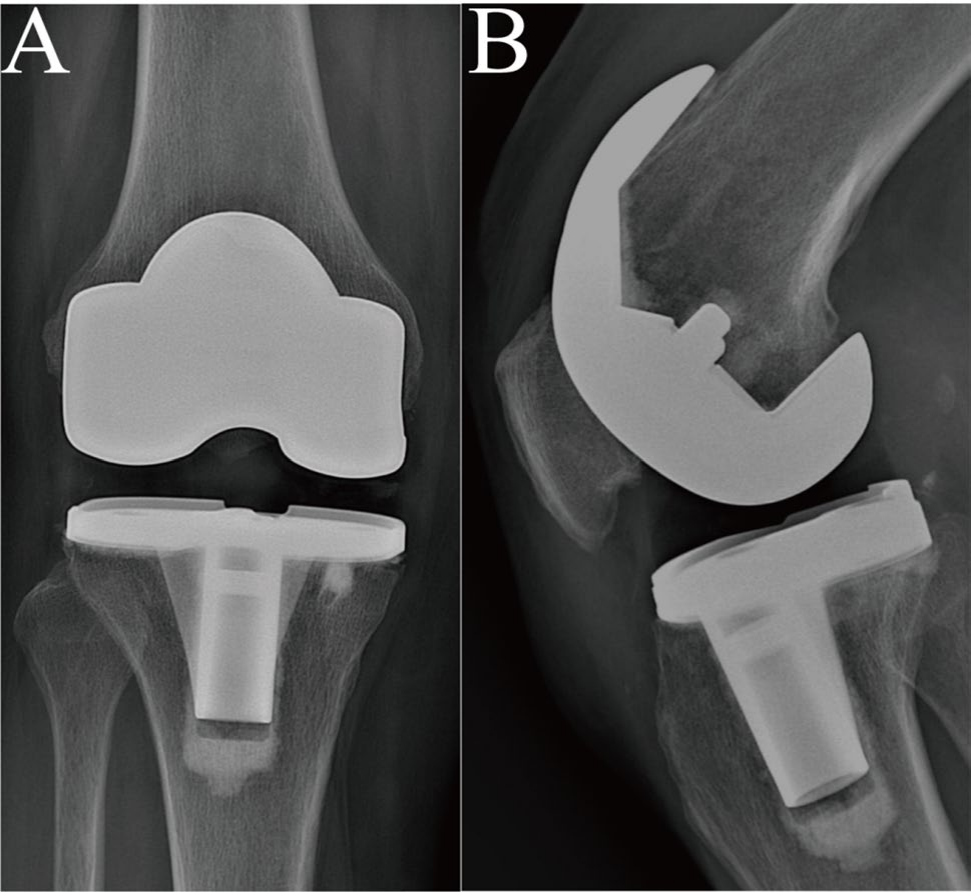
Twelve-month follow-up radiographs after revision total knee arthroplasty (TKA). **A** Anteroposterior view: The revision TKA components are well-positioned with satisfactory mechanical alignment. No radiolucent lines or loosening are observed at the prosthesis-bone interface. **B** Lateral view: The implant maintains appropriate sagittal alignment, with no evidence of component migration, wear, or periprosthetic lucency

## Discussion

### Pathophysiological mechanism: ACL failure due to RA

This case report describes a unique mechanism of late bearing dislocation following mobile-bearing UKA: new-onset RA after a previously successful procedure, leading to chronic inflammatory erosion and functional failure of the ACL, which subsequently induced dislocation of the mobile bearing.

RA is characterized by proliferative synovitis that forms invasive pannus, which not only destroys cartilage and bone but also erodes soft tissues such as ligaments and tendons [10]. Intraoperative findings in this case provided direct visual evidence of this process: the intercondylar notch was filled with hyperplastic synovium, and the ACL was encased and infiltrated by inflammatory tissue, resulting in marked ligamentous attenuation and complete loss of tension. The intraoperative finding of inflammatory synovium directly encasing and infiltrating the ACL strongly implicates RA as the important mechanism.

Although the intraoperative findings strongly suggest that ACL failure is caused by newly developed invasive pannus in RA, the possibility of multiple contributing factors should still be considered. The ACL in mobile-bearing UKA bears higher physiological and mechanical demands than that in normal knee joints. This sustained mechanical load is likely to render the ligament more susceptible to enzymatic degradation and inflammatory attacks from RA synovitis. We propose that ACL failure is the result of the combined effects of mechanical and inflammatory factors. Specifically, the mobile-bearing UKA prosthesis imposes greater mechanical requirements on the ACL, placing the ligament in a persistent “high-load” state even before the onset of RA [11, 12]. This state does not directly damage the ligament but reduces its resistance to injury. Following the development of RA, the inflammatory synovial tissue targets this “weakest link,” accelerating ligament erosion and laxity, which ultimately leads to liner dislocation. Nevertheless, considering the absence of trauma history, acute exacerbation of a chronic course, and pathological findings confirming synovial tissue invasion of the ligament, RA can be clearly identified as the primary determining etiology in this series of chain reactions.

### Biomechanical dependency of mobile-bearing UKA on ligament integrity

The design rationale of mobile-bearing UKA relies heavily on intact knee ligament function, particularly that of the ACL. Goodfellow and O’Connor, the designers of the Oxford UKA, systematically described the role of the ACL in mobile-bearing designs, emphasizing that a functionally intact ACL is essential for facilitating femoral rollback, preventing anterior tibial translation, and maintaining the polyethylene bearing within a safe range of motion [7].Subsequent biomechanical studies have further elucidated this relationship. Mancuso et al. demonstrated that ACL deficiency is a significant risk factor for bearing dislocation and subluxation following mobile-bearing UKA [5]. The ACL-deficient knee exhibits altered kinematics, including increased anterior tibial translation and abnormal femoral rollback, which directly stresses the mobile bearing and predisposes it to dislocation [7, 11]. A finite element analysis study further quantified this effect. The study established UKA models incorporating ACLs of different diameters and found that when a specific load is applied, the stress on the ACL increases significantly with an increase in its diameter. Meanwhile, the ACL can also reduce the stress on the polyethylene liner to a certain extent, which may alleviate the wear of the polyethylene liner and thereby lower the risk of liner dislocation [11]. In the context of inflammatory arthritis, progressive ligamentous erosion creates an acquired ACL-deficient state, with biomechanical consequences analogous to those of traumatic or degenerative ACL insufficiency.

The intraoperative finding of complete functional loss of the ACL in our patient illustrates that inflammatory damage can render the ligament biomechanically incompetent even before structural disruption becomes complete. This insight may explain why some patients with inflammatory arthritis experience instability without frank ligament rupture.

### Comparison with previously reported cases

The design essence of the mobile-bearing Oxford UKA lies in its low contact stress, which is highly dependent on the integrity of the ligaments, especially the anterior cruciate ligament. Traditionally, rheumatoid arthritis has been considered a contraindication to unicompartmental knee arthroplasty because synovitis may involve multiple compartments. However, few reports in the literature have described the mechanism of late-onset bearing dislocation caused by newly developed rheumatoid arthritis after surgery.

In this case, the pathophysiological chain is clear: newly developed rheumatoid arthritis triggered aggressive inflammatory synovitis. Intraoperatively, synovial pannus was observed to encase and erode the ACL, resulting in substantial injury and functional loss of the ligament. Failure of the anterior cruciate ligament disrupted the knee joint’s screw-home mechanism, depriving the tibiofemoral joint of constraint during motion, and ultimately leading to bearing dislocation under minor external force. The significance of this pathophysiological mechanism is further supported by a comparative review of the literature.

Hayakawa et al. reported a similar case of failure after UKA associated with rheumatoid arthritis: the patient developed knee pain due to RA at 7 years and 9 months postoperatively [13]. Although erosion of the lateral femoral and tibial condyles was found intraoperatively, both the anterior and posterior cruciate ligaments remained intact, and only posterior migration of the polyethylene bearing without dislocation was observed.

A notable difference between the two cases in clinical outcome is the status of the anterior cruciate ligament. In the case reported by Hayakawa et al., the anterior cruciate ligament remained intact; despite lateral compartment degeneration, the bearing only migrated posteriorly without dislocation. In contrast, in the present case, aggressive synovitis caused substantial injury and functional loss of the anterior cruciate ligament, ultimately resulting in bearing dislocation. This comparison suggests that the functional integrity of the anterior cruciate ligament plays a critical role in maintaining the stability of the mobile-bearing insert.

### Preoperative Screening strategy for inflammatory arthritis

Through this case, we aim to emphasize that preoperative screening for inflammatory arthropathies should be performed in patients undergoing primary UKA. However, its implementation must be rigorously evaluated from the perspectives of cost-effectiveness and clinical practicality. We acknowledge that universal anti-CCP antibody testing for all UKA candidates yields a very low diagnostic positive rate and would impose a significant economic burden on the healthcare system. Therefore, we advocate a risk-stratified two-step approach designed to balance diagnostic sensitivity with economic sustainability.

First-line testing—consisting of routine inflammatory marker assays and detailed collection of rheumatological medical history—is low-cost, highly accessible, and can be seamlessly integrated into existing preoperative workflows. Its core value lies in its high negative predictive value, which enables the effective exclusion of overt inflammation in the majority of patients. This initial screening step ensures that more expensive and specific second-line tests (e.g., rheumatoid factor testing, anti-CCP antibody testing) are reserved for a small subset of high-risk individuals, namely patients with abnormal baseline screening results or specific risk factors such as elevated routine inflammatory markers and a family history of autoimmune diseases. This targeted approach increases the pre-test probability, thereby enhancing the cost-effectiveness of advanced serological testing.

### Limitations

This study has several limitations. First, as a single case report, the findings are hypothesis-generating and cannot establish causality or generalizability. Second, neutrophil percentage, white blood cell count and differential were not performed, nor was alpha-defensin testing used to rule out infection. These shortcomings compromised the completeness of preoperative diagnostic evidence for PJI, and this limitation warrants improvement in future studies. Third, the long-term durability of the revision TKA in the setting of RA remains unknown and requires continued surveillance. Despite these limitations, the detailed clinical, radiographic, intraoperative, and histopathological documentation provides robust evidence for the proposed pathomechanism and offers valuable lessons for clinicians managing similar cases.

## Conclusions

The etiology of bearing dislocation following mobile-bearing UKA is multifaceted. During both preoperative evaluation and the management of late postoperative complications, maintaining a high index of suspicion for inflammatory joint disease is essential. Implementing appropriate serological screening and adopting a multidisciplinary approach that incorporates rheumatologic care can optimize long-term patient outcomes.

## Data Availability

Data sharing is not applicable to this article as no datasets were generated or analysed during the current study.

## Abbreviations

ACL: Anterior cruciate ligament
PJI: Periprosthetic joint infection
UKA: Unicompartmental knee arthroplasty
RA: Rheumatoid arthritis
anti-CCP: Anti-cyclic citrullinated peptide
CRP: C-reactive protein
ESR: Erythrocyte sedimentation rate

## References

[1] Koh YG, et al. Anatomy-mimetic design preserves natural kinematics of knee joint in patient-specific mobile-bearing unicompartmental knee arthroplasty. Knee Surg Sports Traumatol Arthrosc. 2020;28(5):1465–72. 31123794 10.1007/s00167-019-05540-0

[2] Sun X, et al. Bearing dislocation of mobile bearing unicompartmental knee arthroplasty in East Asian countries: a systematic review with meta-analysis. J Orthop Surg Res. 2021;16(1):28. 33413535 10.1186/s13018-020-02190-8 PMC7791981

[3] Jiao X et al. In Vitro application of a wireless sensor in flexion-extension gap balance of unicompartmental knee arthroplasty. J Vis Exp. 2023(195):10.3791/64993. 10.3791/64993 37212574

[4] Vajapey SP, Alvarez PM, Chonko D. Bearing failure in a mobile bearing unicompartmental knee arthroplasty: an uncommon presentation of an implant-specific complication. Arthroplasty. 2021;3(1):16. 35236477 10.1186/s42836-021-00073-9 PMC8796517

[5] Mancuso F, et al. Medial unicompartmental knee arthroplasty in the ACL-deficient knee. J Orthop Traumatol. 2016;17(3):267–75. 27160183 10.1007/s10195-016-0402-2 PMC4999376

[6] Cao H, et al. Patient-reported outcomes in Chinese rheumatoid arthritis patients: a systematic review and meta-analysis. Chin Med J (Engl). 2021;135(4):471–3. 34354004 10.1097/CM9.0000000000001582 PMC8869620

[7] Goodfellow J, O’Connor J. The anterior cruciate ligament in knee arthroplasty. A risk-factor with unconstrained meniscal prostheses. Clin Orthop Relat Res. 1992(276): p. 245–52. 1537161

[8] Kozinn SC, Scott R. Unicondylar knee arthroplasty. J Bone Joint Surg Am. 1989;71(1):145–50. 2643607

[9] Deckey DG, et al. Rheumatoid Arthritis Is Not a Contraindication to Unicompartmental Knee Arthroplasty. J Arthroplasty. 2024;39(8):2003. –2006.e1. 38428692 10.1016/j.arth.2024.02.067

[10] Ostrowska M, et al. Cartilage and bone damage in rheumatoid arthritis. Reumatologia. 2018;56(2):111–20. 29853727 10.5114/reum.2018.75523 PMC5974634

[11] Ou D, et al. Anterior cruciate ligament injury should not be considered a contraindication for medial unicompartmental knee arthroplasty: Finite element analysis. PLoS ONE. 2024;19(3):e0299649. 38470904 10.1371/journal.pone.0299649 PMC10931476

[12] Kono K, et al. Cruciate ligament force of knees following mobile-bearing unicompartmental knee arthroplasty is larger than the preoperative value. Sci Rep. 2021;11(1):18233. 34521921 10.1038/s41598-021-97655-z PMC8440682

[13] Hayakawa K et al. Revision total knee arthroplasty due to rheumatoid arthritis after unicompartmental knee arthroplasty: A Case Report*.* 2016.

